# Factors affecting retention in the Philippine National Rural Physician Deployment Program from 2012 to 2019: a mixed methods study

**DOI:** 10.1186/s12913-021-07219-0

**Published:** 2021-11-05

**Authors:** Erika Louise L. Flores, Edric Matthew R. Manahan, Miguel Paulo B. Lacanilao, Isabella Ma. Beatriz T. Ladaw, Mico Martin B. Mallillin, Nikolai Thadeus Q. Mappatao, Juan Alfonso Leonardia, Veincent Christian F. Pepito

**Affiliations:** 1 School of Medicine and Public Health, Ateneo de Manila University, Ortigas Ave., Pasig City, Philippines; 2 Deutsche Gesellschaft für Internationale Zusammenarbeit (GIZ), Makati City, Philippines

**Keywords:** Retention, Doctors to the barrios, Rural health, Health workforce, Human resources for health, Philippines, Health services, Public health

## Abstract

**Background:**

To address the maldistribution of healthcare providers and the shortage of physicians in geographically isolated and disadvantaged areas of the Philippines, the Philippine National Rural Physician Deployment Program, or more commonly known as the Doctors to the Barrios (DTTB) program was established in 1993. However, as of 2011, only 18% of the DTTBs chose to stay in their assigned municipalities after their two-year deployment, termed retention. This study aims to identify the individual, local, work, national, and international factors affecting the retention of DTTBs in their assigned communities after their two-year deployment.

**Methods:**

A descriptive, mixed-methods, explanatory design was used. For the quantitative part, the modified and updated Stayers Questionnaire was given to all current DTTBs present in a Continuing Medical Education session in the Development Academy of the Philippines. Descriptive statistics were then presented. For the qualitative part, individual, semi-structured key informant interviews were conducted in-person or via phone with current and alumni DTTBs from 2012 to 2019. Proceedings of the interviews were transcribed, translated, and analyzed thematically.

**Results:**

102 current DTTBs participated in the quantitative part of our study, while 10 current and former DTTBs participated in the interviews. Demographic factors and location, personal beliefs, well-being, friends and family dynamics, and perceptions about work were the individual factors identified to affect retention. Social working conditions, career development, and infrastructure, medical equipment, and supplies were among the work factors identified to affect retention. Geography, living conditions, local social needs, and technology were among the local factors identified to affect retention. Compensation, the recently signed Universal Healthcare Law, and Safety and Security were identified as national factors that could affect retention. International factors did not seem to discourage DTTBs from staying in their communities.

**Conclusions:**

A host of individual, work-related, local, national, and international factors influence the DTTB’s decision to be retained in different, complex, interconnected, and dynamic ways. We also identified implementation issues in the DTTB program and suggested interventions to encourage retention.

**Supplementary Information:**

The online version contains supplementary material available at 10.1186/s12913-021-07219-0.

## Introduction

There is a significant shortage of human resources for health in low and middle-income countries, including the Philippines [1–4]. As of 2011, there are only around two physicians per 10,000 population in the country [5]. Further aggravating the problem is the maldistribution of healthcare providers, including doctors, throughout the country; most of them being concentrated in urban areas. As a result, far-flung areas in the country have very few healthcare providers, with some areas only having one physician per 20,000 population [4–6]. To address the shortage and maldistribution of healthcare providers, it is essential not just to recruit additional healthcare workers, but also to ensure their equitable distribution throughout the countries affected by these crises [7, 8]. To address both problems in the Philippines, the country’s Department of Health (DOH) formulated the Philippine National Rural Physician Deployment Program, more commonly known as the Doctors to the Barrios (DTTB) program.

Implemented since 1993, the DTTB program aims to attract competent, committed and community-oriented medical doctors to render health services in geographically isolated and disadvantaged areas (GIDA) throughout the country which lack physicians [9]. The physicians serve for two years in their assigned areas where they were given competitive pay (the current gross monthly compensation is around Php 90,000 or USD 1870), additional hazard pay for doctors assigned to areas with humanitarian conflict, scholarship for a Master’s degree in Public Management – Major in Health Systems Delivery (MPM-HSD) from the Development Academy of the Philippines, and benefits described in the Magna Carta for Public Health Workers such as subsistence and laundry allowance, housing and insurance, among others. After their two-year deployment, they could either practice as an epidemiologist, do public health research, undergo residency training in public or private hospitals, or remain in their assigned municipality; the last outcome would be the most ideal to ensure that these GIDAs would have at least one physician in their locality [9–11].

However, retention in the program, or DTTBs’ choosing to stay and work in the localities where they were assigned to after the two-year contract, has been a continuing challenge. From 1993 to 2011, only 18% had chosen to stay in the program due to various reasons, such as personal satisfaction, working environment, and career development [12]. However, many developments, such as increase in coverage and utilization of internet and social media [13], and incidents where two current or former DTTBs have been killed in the line of duty, have occurred since then [14]. Furthermore, the previous study has not looked at the psychosocial experiences of these health practitioners while deployed in the rural municipalities, and the impact of international factors on retention. These issues highlight the need to revisit the factors affecting retention among DTTBs to help in the development of effective interventions to address the needs of the DTTBs and to encourage them to continue working in their assigned rural areas. Thus, this study aimed to identify the enabling factors and barriers affecting the retention of DTTBs in their assigned locality after their two-year deployments.

## Methods

### A. Study design, population, and data collection

A descriptive, explanatory design was used in this study. We started with a quantitative design to measure the proportion of DTTBs experiencing implementation issues which could affect retention. To further explain, expand, and give context to the findings from the quantitative survey, we followed-up with a qualitative design. The latter design also allowed us to solicit recommendations to improve the Program from the DTTBs themselves [15].

For the quantitative part, we conducted a survey with all currently deployed DTTBs present at the DAP Convention Center, Tagaytay City, during a Continuing Medical Education (CME) session in May 2019. The survey utilized a self-administered questionnaire (SAQ), specifically an updated version of the validated modified Stayers Questionnaire by Leonardia et al. [12], which was pre-tested on 15 doctors with at least one year of experience working in different Philippine provinces and updated accordingly [16]. The final version of the Questionnaire used in the study is attached as Additional file 1. Prior to the distribution of the SAQ, the DTTBs were oriented by the researchers and given informed consent forms. After the DTTBs have given their consent to participate, the respondents were given 45 min to complete the questionnaire; after which, they dropped it in a secure drop box to ensure their anonymity.

For the qualitative data, both currently deployed and former DTTBs from 2012 to 2019 were interviewed. The current DTTBs were recruited through the CME session while the former DTTBs were recruited through referrals by other former DTTBs. The number of interviewees was based on a recommendation by Creswell and Morse, to have at least six participants to ensure saturation [17–19]. The collection of qualitative data lasted from May to September 2019 and used the pre-tested interview guides (attached as Additional file 2). This entailed key informant interviews using a semi-structured interview guide with respondents in the DAP or in different venues in Metro Manila at their choice and comfort. However, since some of the participants were currently working in the provinces, they also have the option to participate in the study through a phone interview. Regardless of whether the interview was conducted in person or over the phone, the proceedings of the interview were audio-recorded, then transcribed. The average interview time was one hour.

Prior to conducting the study, we sought approval from the DOH – Health Human Resources Development Board, the government agency overseeing the DTTBs. The research protocol was approved by the technical review board and ethics committee of the Ateneo de Manila University School of Medicine and Public Health.

### B. Data analysis

The quantitative data from the SAQs were encoded through Epi Info 7 [20] and analyzed through Microsoft Excel [21]. Frequencies and proportions were used to describe categorical data, and measures of central tendency and dispersion were used to describe continuous data.

The transcribed interview proceedings, together with the responses to the open-ended questions in the SAQ, were translated into English and were analyzed thematically using NVivo [22]. Each open-ended questionnaire and transcription were coded by at least two researchers to minimize any bias in the analysis. Standardized codes, based on the literature and conceptual framework, were used (e.g., well-being, technology, compensation). Emergent codes were also considered. In the event of disagreement between coders, the researchers discussed thoroughly to agree on which sub-theme and/or main theme these codes belonged.

### C. Conceptual framework

The themes used in this paper are based on a conceptual framework adapted from Lehmann et al. [23]. This framework theorizes that a rural health worker’s decision to stay in or leave a job relies on the complex interplay of retention factors within the individual, working, local, national, and international environments. Briefly, the individual environment refers to factors that work at the personal level such as demographic factors and well-being. The working environment pertains to factors such as working conditions, infrastructure and equipment, and relationship with colleagues. Local environment pertains to how their living conditions affect retention. National environment pertains to how government policies and events affect their retention. Lastly, the international environment pertains to how factors beyond the country where the health worker resides affect their tendency to stay in their current community [23–25]. Organizing retention factors according to environments make it easier for policymakers and other relevant parties to pinpoint areas for intervention without losing sight of the whole retention picture [23].

## Results

### A. Description of study participants

A total of 102 out of 108 (94.4%) DTTBs answered the SAQ; six (5.6%) DTTBs did not participate due to their absence during the CME session. Three participants had missing data on work environment, local environment, and national environment factors. Most participants were female, less than 30 years old, single, spent their youth in rural areas, educated in medical schools in Metro Manila, and do not have any return service obligation (Table 1). For the interviews, a total of ten DTTBs participated; one alumnus each from Batch 2012 and Batch 2014, two alumni from Batch 2015, and six from Batch 2017. Nine out of 10 interview participants were male.

**Table 1:** Demographics of the SAQ participants (*n* = 102)

Demographic Factor		Number (%)
**Sex**	Male	40 (39.2%)
	Female	62 (60.8%)
**Age**	Mean Age (Std. dev)	28.68 years old (± 2.77)
	Min/Max	24/40 years old
**Civil Status**	Single	86 (84.3%)
	Married	16 (15.7%)
**Permanent Residence**	Within NCR (National Capital Region)	16 (15.7%)
	Outside of NCR	85 (83.3%)
	Missing information	1 (1.0%)
**Dependents**	With	33 (32.4%; 1.85 dependents per DTTB)
	Without	69 (67.6%)
**Spent their youth in rural areas**	Yes	63 (61.8%)
	No	39 (38.2%)
**Rural upbringing influenced their decision to apply for DTTB**	Yes	49/63 (77.8%)
	No	14/63 (22.2%)
**Medical School Education**	Within NCR	60 (58.8%)
	Outside of NCR	42 (41.2%)
**Return Service Agreement**	With	37 (36.3%)
	Without	65 (63.7%)

### B. Individual environment

#### Demographic factors and location

Three out of ten interviewees highlighted marital status and proximity to hometown (i.e., being married and far from home) made staying less desirable:

"*My area is 1 hour and 30 minutes away by boat, 3 hours away by land travel from my area of residence. I just got married, so the distance is really a factor in assessing (whether to stay or not). That is one of the factors I’m looking into if I’m planning to extend." (DTTB # 2)*

#### Personal beliefs

Most respondents found fulfillment in serving in their respective areas (94%) and found work as meaningful and stimulating (83%). The majority of the interviewed DTTBs weighed in service to country and personal growth on their decision to be retained. Regarding service to the country, the DTTBs said a “higher purpose” – the need to serve and impact the public health system – brought them back to their community and would make them stay.

Meanwhile, according to one DTTB, the current generation values personal growth more than rooting down, hence fewer DTTBs stay now compared to those from older batches. Another respondent mentioned that one should become and remain a DTTB because of the benefit of holistic personal development.

#### Well-being

Well-being was repeatedly discussed by the majority of the interviewed DTTBs. Central to well-being was the experience of burnout, which was managed through various coping strategies. Mental health, particularly depression, and the sense of stability (i.e., family and financial stability) also emerged as influencing factors on the DTTBs’ decision to stay.

The interviewed DTTBs highlighted the following coping strategies: [a] taking breaks, [b] having a support system, [c] emotional detachment, [d] problem prioritization, and [e] human resource management. The DTTBs discussed taking breaks by emphasizing the importance of leisure and “returning to normalcy” (e.g., going to the mall) to temporarily escape from the stresses of work. Their support system consists of other currently deployed DTTBs, DTTB alumni who frequently become mentors, friends, and “family”. Emotional detachment allows the DTTB to go through the two-year deployment without expending all emotional energy. Problem prioritization involves discerning then choosing the winnable battles and letting go of the futile ones. Finally, managing work relationships involves knowing how to approach or interact with people in such a way that they can be swayed to aid the DTTB in reaching his/her goals for the municipality.

One interviewed DTTB was diagnosed with major depressive disorder (MDD) during his tenure as a DTTB, while another DTTBs discussed how depressive feelings interfered or could possibly interfere with their work. Feelings of hopelessness, loss of purpose, and blunted affect can discourage functionality in society, thus, discourage retention:

"*I questioned why I was doing this. I like what I’m doing but it’s not rewarding in my opinion at that time in my area ... It’s like I’m constantly depleted. I feel like I’m always giving to the community, but, in the end, I become depleted. It looks like you are doing good, but it seems like nothing good is happening to you." (DTTB # 6)*

#### Friends and family dynamics

Around three quarters of respondents reported that their families supported their decision to become a DTTB (73%). This is remarkable considering that relationships with friends and family, particularly having a significant other, children, nostalgia for family, and actual family dynamics were important in the DTTBs’ decision to stay or leave.

One DTTB said that distance from a significant other became an issue in their relationship. Another DTTB said that she would consider staying because it is easier to start a family as a DTTB rather than as a resident physician in a hospital, given that work arrangements (e.g., leave credits, relaxed pace, fewer demands, thus, more personal time) are more flexible as a DTTB. This same DTTB went on to share that it would be good for her child to see her as a DTTB, as it would educate the child about the public health situation of the Philippines.

Furthermore, one DTTB talked about how he missed his mother cooking for him. Some DTTBs also added that a supportive and less demanding family encourages retention. Conversely, a high-strung, excessively worried family makes retention less appealing.

#### Perceptions about work

Two perceptions were particularly influential in the DTTBs’ decision to stay or leave: the multiple roles of a DTTB, and the “I wish I could do more” mentality.

The majority of the interviewed DTTBs stated that they found their workload to be heavy and tiring due to the multiple roles that they must adopt: physician, public health manager, and MPM-HSD student. The administrative work is particularly difficult for them because they feel that they were not adequately trained by their medical school and the DOH for this role. In some cases, such workload has led to burnout and ultimately decreased well-being. Nevertheless, the workload has also led to professional skills development, which encourages the DTTBs to stay.

Many of the interviewed DTTBs feel that they were not able to fulfill their goals (e.g., complete a project) as a DTTB. Consequently, the DTTBs think “I wish I could do more,” which makes them want to stay longer in their localities to fulfill these goals.

### C. Work-related environment

**Fig. 1:**
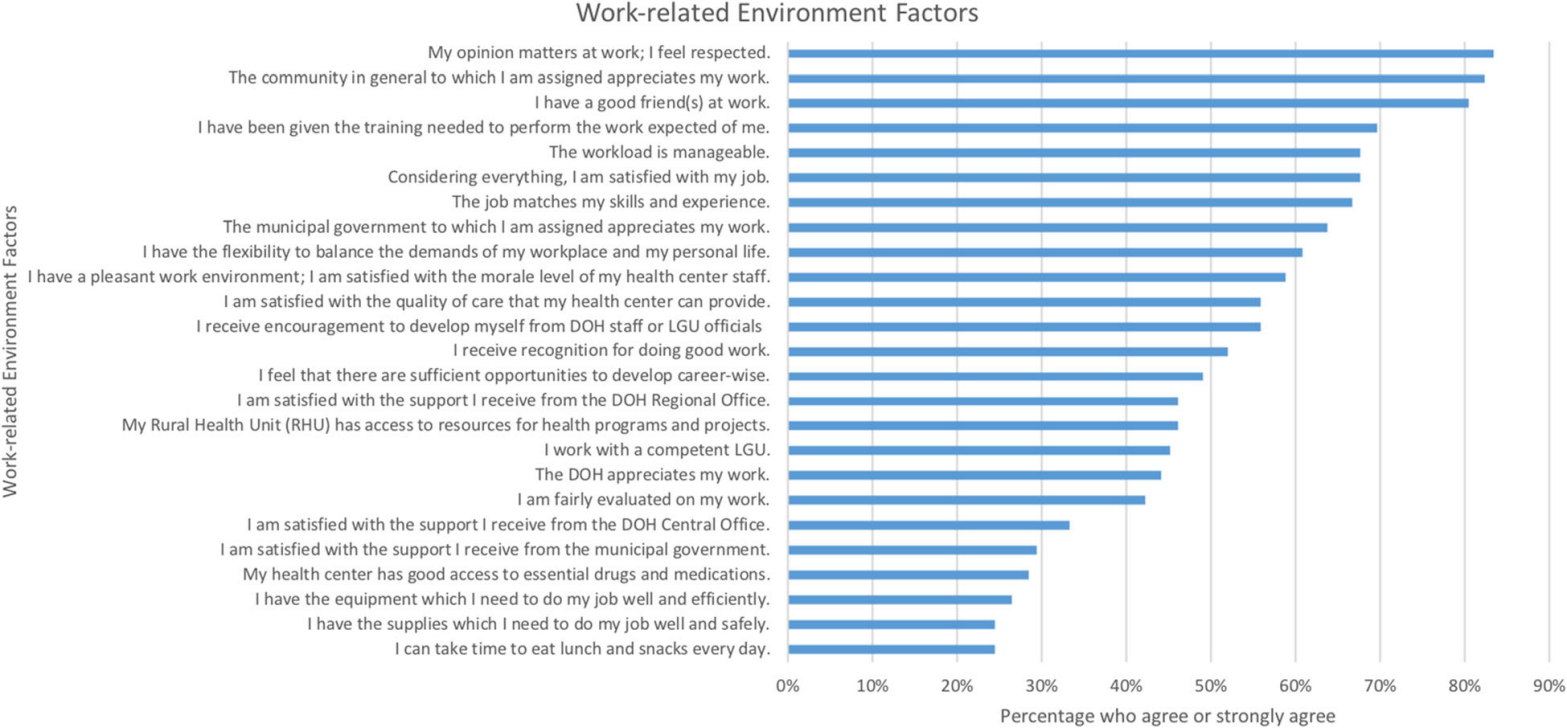
Percentage of DTTBs, who agree or strongly agree with work-related factors (*n* = 102)

Many DTTBs feel that their opinion is valued and respected (83%), that their community appreciates their effort (82%), and that they have good friends at work (80%). However, only a quarter of DTTBs reported having adequate supplies (25%) and time to eat (25%). Less than 30% feel that they are satisfied with the support of their host municipal government and less than 35% feel satisfied with the support of the DOH. Poor social working conditions and perceived lack of support could adversely affect retention (Fig. 1).

#### Social working conditions

Among all the work-related influencers, social working conditions (i.e., related to people like the staff and leadership) emerged as the most talked-about factor for the majority of the interviewed DTTBs. Four main conditions served to shape the DTTBs’ thoughts regarding staying in their assigned municipalities: [a] the organization of the DOH-Local Government Unit (LGU)-DTTB system, [b] informal social networks, [c] relationship with subordinates, and [d] relationships with patients.

The haphazard interaction between DOH, the LGU, and the DTTB are characterized by inefficient communication (e.g., faulty emergency referral system to tertiary hospitals), and disparate expectations regarding the role of the DTTB in the rural health unit (i.e., municipal health officer or rural health physician). Furthermore, the differences in goals for the municipality between the LGU and DTTB, and feelings of lack of DOH and LGU support make it difficult for DTTBs to fulfill their tasks and help the municipality, leading to stress and discouraging retention.

Being able to form and navigate informal social networks (i.e., professional connections not defined by the official organizational structure) helps in accomplishing work or making it easier. These include dealing with the health center staff, local government officials, DOH officials, and other local health stakeholders on a day-to-day basis. Successful informal social networks and having good relationships and being appreciated by patients make retention more feasible. On the other hand, some DTTBs talked about health center staff whom they felt were unprofessional, under-skilled, insubordinate, insufficient in number, unnecessarily absent and poorly motivated, among other reasons. These unsatisfactory relationships with health center staff discourage retention. Moreover, compromising work-life balance due to the workload makes retention less appealing.

#### Career development

The availability or lack of opportunities leading to career growth in the assigned municipalities influence the DTTBs decision to either stay or leave, respectively. Some DTTBs, however, were more aware of career opportunities than others. Additionally, the majority of the interviewed DTTBs felt that they have learned from the DTTB experience, particularly with regards administration, human resource management, and inventory management, among other skills, which they feel would help them throughout their careers.

#### Infrastructure, medical equipment, and supplies

The DTTBs think that infrastructure, medical equipment, and supplies in the community are insufficient, inadequate, and substandard; nevertheless, these were not considered enough to discourage retention. They merely referred to these shortcomings as “challenging”.

### D. Local environment

**Fig. 2:**
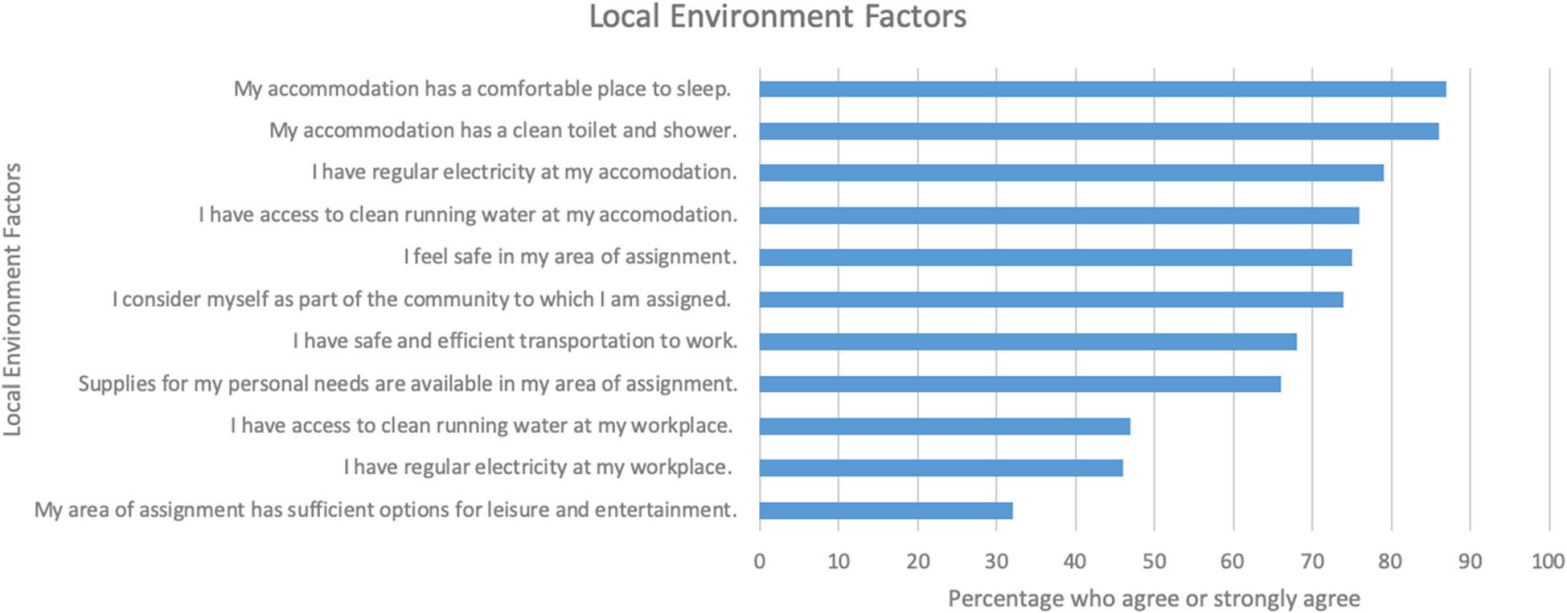
Percentage of DTTBs that agree or strongly agree with local factors (*n* = 102)

Many DTTBs report that they have accommodations that are comfortable for sleeping (85%) and have a clean toilet and shower (84%). However, less than half of them have clean running water (46%) and regular electricity (45%) in their workplace. Lastly, only 31% have sufficient options for leisure and entertainment; such local environment factors also play a role in their decision to stay in their locality (Fig. 2).

#### Geography

Only three out of ten interviewees talked about this factor, largely because of the variations in geography across the different municipalities. Nonetheless, geography is a noteworthy influencer on the decision to stay because it shapes work logistics (e.g., transportation and supplies delivery), and thus, the DTTB’s opportunity and ability to manage patients. Losing the sense of agency to help patients discourage retention:

"*There was a time that they brought a patient to me in the middle of the night and there was no electricity, no oxygen. The patient was highly distressed, but we could not transport him because number one, he did not want to be transported, and number two, there was no boat [to cross the sea]." (DTTB # 9)*

#### Living conditions

Despite it being stipulated in their deployment contract, there are sometimes no set accommodations and financial aid for living expenses for DTTBs. This is exacerbated by lack of food choices, scarcity of electricity and potable water, and limited transportation (i.e., once a day boat ride). Such unsatisfactory living conditions may discourage retention.

#### Local social needs

Due to their daily workload, there is evidently less time for DTTBs to spend with their close friends and family. This is exacerbated by the lack of options for leisure and entertainment, potentially discouraging retention.

#### Technology

Because DTTBs are often assigned to areas far from friends and family, they rely on technology to communicate with them. Communication devices/media used by the DTTBs for this purpose include mobile phones, social media applications (e.g., Facebook®, Twitter®), and other online telecommunications applications like Viber®, Messenger®, Skype®, and FaceTime®. Therefore, the availability of a cellphone signal and internet connectivity are among the main considerations of DTTBs on whether to stay in their area after deployment. Having a poor cellphone signal or internet connection may discourage retention.

### E. National environment

**Fig. 3:**
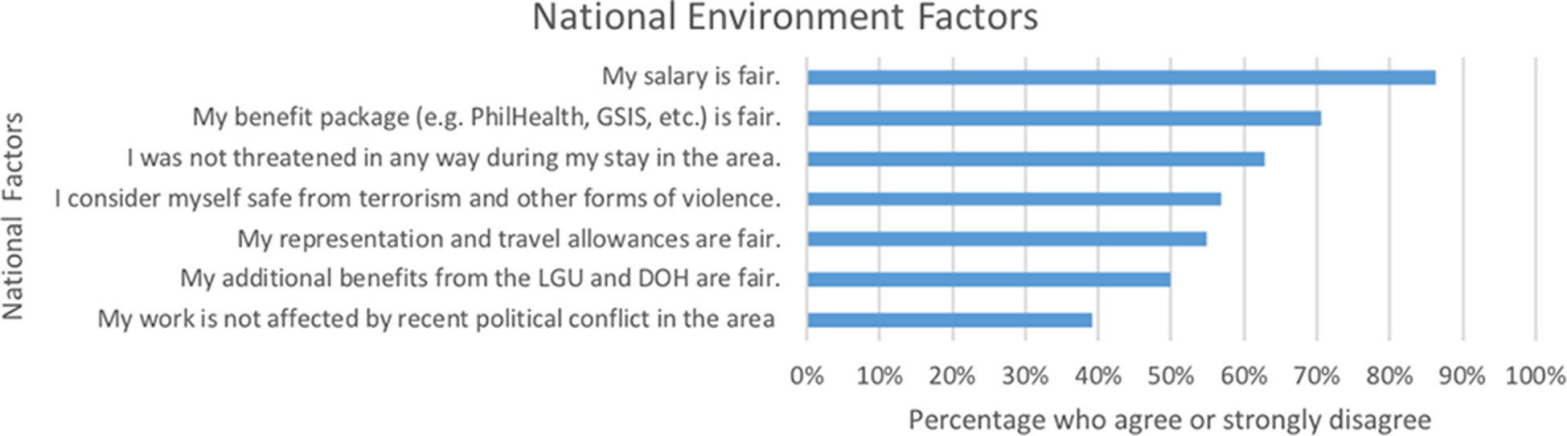
Percentage of DTTBs that agree or strongly agree with national factors (*n* = 102)

Majority of the respondents believe that their wages (86%) and benefit packages (71%) are fair. However, only few respondents report that their work is not affected by recent political conflict in their area of deployment (39%), which could discourage retention (Fig. 3).

#### Compensation

Out of all the factors discussed in this study, the DTTBs considered good compensation as one of the most important in their decision to be retained. While most of the DTTBs interviewed mentioned that their salary is high considering that being a DTTB is an entry-level profession, some still do not feel that this salary is commensurate to the amount of work that the DTTBs do. Additionally, there are often delays in the processing of salaries of the DTTBs, sometimes up to four months. While this issue has been mentioned several times to the central DOH office, several DTTBs report that no tangible solutions have been made so far.

Furthermore, no executive medical check-ups are given to the DTTBs. This is despite the DTTBs’ mentioning that they think it is essential for them to have these medical consultations, especially since they are exposed to several infectious diseases in the community such as tuberculosis and malaria. The DTTBs also complained that vaccinations are not made available to them.

#### Universal health care (UHC) act

The Universal Health Care Act seeks to give Filipinos increased access to health-related services by enrolling all Filipino citizens into the National Health Insurance Program of the Philippine Health Insurance Corporation. The UHC act was considered as an important factor in the DTTBs’ decision to be retained as this may eventually lead to higher priority for DTTBs and their respective health projects. A former DTTB cited that he may have stayed longer in his area if the UHC act had been implemented while he was still in the program, since this may make it easier to attain the DOH-set health indicators and goals for the community. Other current DTTBs also talked about how the full implementation of the UHC act would facilitate their work and overall experience as DTTBs (e.g., gain more respect from staff, local government officials, etc.), encouraging retention. Another said that being a DTTB allows her to make contributions to the UHC act. Hence, she can impact the Philippine public health system as a DTTB, which makes her want to stay.

#### Safety and security

Being assured that they are safe while being deployed to the community is one of the top priorities of the DTTBs for retention in the program. However, according to them, there is no existing safety and security protocol from the DOH in instances of social unrest/conflict within their area. This protocol is important, especially since many of the DTTBs are assigned to rural areas with terrorists and/or insurgents. DTTBs are also often caught in between conflicting political parties, which affects their safety and ability to work. Nevertheless, some areas were noted to be free from socio-political unrest. Some also noted the lack of or delayed monitoring of DTTBs in the communities, especially in times of natural disasters which commonly affect the country.

### F. International environment

Opportunities such as higher rates of remuneration, more satisfying working conditions, higher quality of life, a safer working environment, and better educational and career development opportunities were the common reasons as to why DTTBs would prefer to work abroad. However, some DTTBs identify the current Philippine health system to be a great avenue to practice public health, encouraging retention in the program.

## Discussion and recommendations

A host of individual, work-related, local, national, and international factors influence the DTTB’s decision to be retained in different, complex, interconnected, and dynamic ways. Some of these factors, such as the sense of fulfillment, being a respected member of the community, social support systems, competitive compensation, career development in their community after their tenure, and enactment of new laws to strengthen public health in the Philippines encourage DTTBs to stay in their communities even after deployment. On the other hand, geographic isolation, burn-out, poor access to electricity, telecommunications, and potable water, lack of support from their host LGUs and from the national government, and safety and security issues hinder DTTBs from staying in their communities after serving their two-year contract. Some of these findings have been corroborated by one scoping review of factors to increase the supply of rural health physicians, but some factors are context-specific and were not touched upon in the review yet [26]. Improving retention of rural health physicians require the implementation of interventions on education, regulation, financial incentives, and professional and personal support [27, 28]. Monitoring and evaluation is also key in ensuring that such interventions work [27].

The multiple roles a DTTB plays, coupled with the emotional and mental demands of the job, could make them susceptible to burnout and depression. As such, there should be mental health interventions from the government, such as regular counselling, debriefing, and coaching and mentoring sessions. This is particularly necessary as there seems to be no mental health interventions to safeguard the mental well-being of DTTBs at present. Such interventions are becoming increasingly important as it has been found that improved coping skills have been associated with rural health worker retention [29]. With the recent signing of the Philippine Mental Health Law [30], hopefully, the DTTBs’ mental health will be prioritized, protected, and actively managed by the DOH such that the DTTBs feel like they are being cared for.

DTTBs, especially those that have good experiences with their host community, often find the two years of DTTB to be insufficient for them to enact their vision for their communities. Thus, it might therefore be favorable to have the option to extend their duration of deployment from the current two years to three. Such an extension will allow DTTBs who want to be retained in their communities a better chance of doing so because they will now become eligible for the next higher position – being a municipal health officer - after meeting the minimum of three years of work experience required for it.

In some cases, there is a mismatch between the priorities of some LGUs, which may not include health, and ensuring that health targets of the DOH are being met, which leaves the DTTB confused and frustrated as to who will be prioritized. As a result, the DTTBs feel that they do not get the support they need to implement public health programs in the community. Lack of supportive leadership and poor relationships with colleagues have been found to contribute to poor retention [23, 26, 31–33]. Because of this, the roles and responsibilities of the DTTB to both DOH and the LGU should be clarified, and the DOH, being the national agency, should play a more active role in ensuring that DTTBs can implement their programs for their communities.

There are also instances where stipulations in the DTTB contract are not met. While it is the responsibility of the government to provide them with accommodation in their place of assignment, sometimes, the DTTB had to look for their own accommodation upon arrival. Some accommodations provided have poor access to quality food, internet, cellphone signal, and even potable water. Unsatisfactory living conditions not only make it challenging for the DTTBs to live in their communities but ultimately, it can interfere with their capacity to work. In the DTTB contract, the DTTBs are supposed to have a living allowance, but this is sometimes not provided. There are also delays in the processing of their salaries, which make living in a community away from home difficult. To improve retention, the DOH should ensure that they satisfy their end of the contract, provide comfortable accommodation to DTTBs, give the requisite living allowances, and ensure that the DTTBs are paid on time.

Natural disasters, together with insurgencies and the recent killings of former and current DTTBs, have highlighted the lack of a safety and security protocol from the DOH for DTTBs and health workers in general. All health workers, particularly DTTBs, can benefit from a protocol outlining what should be done by them, the DOH, and the LGUs during insurgencies, and natural disasters. There should also be a mechanism for DTTBs to report any issues they encounter at work to the DOH which they feel could threaten their lives. There should also be a way for the DOH to respond to these threats to their personnel.

The recently signed UHC law is still beginning to be implemented as of writing; nevertheless, if the law is implemented fully, there are positive implications for retention in the DTTB program. For example, the law provides for the creation of a *National Health Human Resource Master Plan* which guarantees permanent employment and competitive salaries to health workers to ensure continuity in the provision of health programs and services. It also provides for the creation of a *National Health Workforce Support System* which shall support local public health systems like rural health units in addressing human resource needs. Organization of Local Health Systems through provisions such as the *Integration of Local Health Systems into Province-wide and City-wide Health System* may also benefit DTTBs in streamlining efforts from different agencies within their area of assignment [34, 35].

Consistent with previous studies on health care practitioners in international destinations [23], the DTTBs also consider going abroad due to higher rates of remuneration, more satisfying working conditions, higher quality of life, a safer working environment, and better educational and career opportunities. However, it must be considered that some DTTBs are restricted from leaving the country due to their return service agreement which are imposed on graduates of some public medical schools in the country. Furthermore, despite inefficiencies in the health system of the Philippines compared to other countries, the DTTBs would still choose to stay in the country to be an active participant in the development of the country’s public health system. Thus, international factors do not seem significant to discourage DTTB retention.

### Limitations of the study

We only had the opportunity to administer the SAQ to a single batch of DTTBs and had a relatively limited pool of interview participants. This would make our study non-representative throughout the different batches of the program and would limit the generalizability of our findings to DTTBs from 2012 to 2019. Considering the self-report nature of the data collected, there could be significant Hawthorne effect [36], and the validity of the findings of the study are as good as those that are reported by the participants.

## Conclusions

This study found the interplay of various personal, work, local, national, and international environments influencing retention in the Philippine National Rural Physician Deployment Program. Several issues in the implementation of the DTTB program in each of these environments which could discourage retention were noted, and interventions to address these issues were given. Providing an option to prolong the duration of employment from two to three years was suggested to encourage retention. Furthermore, there is a need to create mental health interventions, provide annual physical examinations, and vaccinations to ensure the health and well-being of DTTBs. There is also a need to clarify and enforce the relationship between the DTTB, the LGU and the DOH. There is also a need to provide comfortable accommodation and timely compensation to DTTBs. Lastly, there is a need for a safety protocol for health workers which should describe what DTTBs should do in the case of emergencies or natural disasters and when they encounter threats to their safety. By doing these proposed interventions, we can ensure that DTTBs are cared for and are supported in their undertakings in the community, encouraging them to stay in the communities they serve and addressing inequities in the distribution of physicians in the Philippines.

## Supplementary Information

**Additional file 1.** Modified Stayers Questionnaire

**Additional file 2.** Interview Questions

## Data Availability

The datasets and transcripts used and/or analyzed are available from the corresponding author (vpepito@ateneo.edu) on reasonable request.

## Abbreviations

CME: Continuing Medical Education
DTTB: Doctors to the Barrios
DOH: Department of Health (Philippines)
GIDA: Geographically Isolated and Disadvantaged Areas
LGU: Local Government Unit
MPM-HSD: Master in Public Management major in Health Systems and Development
NCR: National Capital Region
SAQ: Self-Administered Questionnaire
UHC: Universal Health Care
