# Supplementary material for: Factors affecting retention in the Philippine National Rural Physician Deployment Program from 2012 to 2019: a mixed methods study
Source: BMC Health Serv Res. 2021 Nov 5;21:1201. doi: 10.1186/s12913-021-07219-0 (PMC8571874; doi:10.1186/s12913-021-07219-0)
Supplement: Supplementary file 1 — Additional file 1. Modified Stayers Questionnaire [file 12913_2021_7219_MOESM1_ESM.pdf]

## Additional File 1 - Modified Stayers Questionnaire

### PERSONAL INFORMATION SHEET AND CONSENT FORM

**PARTICIPATION IN THIS RESEARCH STUDY IS VOLUNTARY. I am free to decline to participate in this research study, or I may withdraw my participation at any point without penalty. My decision whether or not to participate in this research study will have no influence on my present or future dealings with the Ateneo de Manila University, the local government unit where I am assigned, or with the Department of Health (DOH).**

Signature over printed name: _____________________ Date: _______________

Section I. Personal Information

Please mark the box like this [✓] with a ballpoint pen. If you change your mind just cross out your old response and make your new choice.

| Q. No. | Questions | Please enter or mark your answers |
|---|---|---|
| 1 | What is your gender? | ▢ Male ▢ Female |
| 2 | What is your age? | ▢▢ |
| 3 | What is the **city/municipality** of your permanent residence? | |
| 4 | What is the **province** of your permanent residence? | |
| 5 | What is your marital status?<br>(Please check one category box) | ▢ Single ▢ Widowed<br>▢ Married ▢ Separated<br>▢ Divorced |
| 6 | Do you have children or dependents that you financially support who are living either under or outside your household? | ▢ Yes ▢ No |
| 7 | If **Yes**, how many total dependents? | ▢▢ |
| 8 | Which medical school did you graduate from? | |
| 9 | What is the **city/municipality** of your medical school? | |
| 10 | What is the **province** of your medical school? | |
| 11 | In which year did you finish your medical internship? | ▢▢▢▢ yyyy |
| 12 | What is your DTTB batch number? | ▢▢ |
| 13 | Which **municipality** are you currently assigned to? | |
| 14 | Which **province** are you currently assigned to? | |

| | | | |
|---|---|---|---|
| 15 | Have you previously worked as a DTTB? | ▢ Yes | ▢ No |
| 16 | If yes to what number?, please list down your previous municipalities of assignment | | |
| 17 | Is this your first job since graduating from medical school? | ▢ Yes | ▢ No |
| 18 | What is/are your main reason(s) for joining the DTTB Program? | | |
| 19 | Were you bound to fulfil any form of return service for a scholarship or any other benefit that you might have previously received? | ▢ Yes | ▢ No |
| 20 | Did you stay in your area of assignment or choose to work in another underserved area after graduating from the DTTB program? | ▢ Yes | ▢ No |
| 21 | If YES to what number?, how long did you work as a rural physician | | |
| 22 | If YES, what were the main reason (s) for your choice? | | |
| 23a | Did you spend any part of your youth in a rural area? | ▢ Yes | ▢ No |
| 23b | If YES, where? | | |
| 24a | Do you think that having lived and spent your youth in a rural area influenced your choice to be a rural physician? | | |
| 24b | If YES to what number?, how? | | |

Section II: Individual Environment Factors

*The following questions refer to your "personal factors," characteristics such as age, gender, marital status, place of origin (rural or urban), ethnicity, personal values and beliefs, and even spiritual motivations while working as a DTTB. Please encircle the number that best fits your level of agreement with each statement, using a 5-point scale where 5=strongly agree, 4=agree, 3=neutral, 2=disagree, 1=strongly disagree.*

| No. | **To what extent do you agree with the following statements?** | 5 = Strongly Agree | 4 = Agree | 3 = Neutral | 2 = Disagree | 1 = Strongly disagree |
|---|---|---|---|---|---|---|
| 25 | I find fulfilment in serving my community. | 5 | 4 | 3 | 2 | 1 |
| 26 | My family supports my decision to work as a DTTB. | 5 | 4 | 3 | 2 | 1 |
| 27 | When I come to work, I know what is expected of me. | 5 | 4 | 3 | 2 | 1 |
| 28 | I enjoy working as a DTTB; the work I am doing is meaningful and stimulating. | 5 | 4 | 3 | 2 | 1 |

Section III: Work-related Environment Factors

*The following questions refer to your "professional factors," these refer mainly to job satisfaction, opportunities for career development, and working conditions while working as a DTTB. Please encircle the number that best fits your level of agreement with each statement, using a 5-point scale where 5=strongly agree, 4=agree, 3=neutral, 2=disagree, 1=strongly disagree.*

| No. | **To what extent do you agree with the following statements?** | 5 = Strongly Agree | 4 = Agree | 3 = Neutral | 2 = Disagree | 1 = Strongly disagree |
|---|---|---|---|---|---|---|
| 29 | Considering everything, I am satisfied with my job. | 5 | 4 | 3 | 2 | 1 |
| 30 | My opinion matters at work; I feel respected. | 5 | 4 | 3 | 2 | 1 |
| 31 | I have a good friend(s) at work. | 5 | 4 | 3 | 2 | 1 |
| 32 | The DOH appreciates my work. | 5 | 4 | 3 | 2 | 1 |
| 33 | The municipal government to which I am assigned appreciates my work. | 5 | 4 | 3 | 2 | 1 |
| 34 | The community in general to which I am assigned appreciates my work. | 5 | 4 | 3 | 2 | 1 |
| 35 | The job matches my skills and experience. | 5 | 4 | 3 | 2 | 1 |
| 36 | I receive recognition for doing good work. | 5 | 4 | 3 | 2 | 1 |
| 37 | I receive encouragement to develop myself from DOH staff or LGU officials | 5 | 4 | 3 | 2 | 1 |
| 38 | I am satisfied with the support I receive from the DOH Central Office. | 5 | 4 | 3 | 2 | 1 |
| 39 | I am satisfied with the support I receive from the DOH Regional Office. | 5 | 4 | 3 | 2 | 1 |
| 40 | I am satisfied with the support I receive from the municipal government. | 5 | 4 | 3 | 2 | 1 |
| 41 | I am satisfied with the quality of care that my health center can provide. | 5 | 4 | 3 | 2 | 1 |
| 42 | I am fairly evaluated on my work. | 5 | 4 | 3 | 2 | 1 |
| 43 | I have been given the training needed to perform the work expected of me. | 5 | 4 | 3 | 2 | 1 |
| 44 | I feel that there are sufficient opportunities to develop career-wise. | 5 | 4 | 3 | 2 | 1 |
| 45 | I have a pleasant work environment; I am satisfied with the morale level of my health center staff. | 5 | 4 | 3 | 2 | 1 |
| 46 | The workload is manageable. | 5 | 4 | 3 | 2 | 1 |

| | | | | | | |
|---|---|---|---|---|---|---|
| 47 | I have flexibility to balance the demands of my workplace and my personal life. | 5 | 4 | 3 | 2 | 1 |
| 48 | I can take time to eat lunch and snacks every day. | 5 | 4 | 3 | 2 | 1 |
| 49 | I have the supplies which I need to do my job well and safely. | 5 | 4 | 3 | 2 | 1 |
| 50 | I have the equipment which I need to do my job well and efficiently. | 5 | 4 | 3 | 2 | 1 |
| 51 | My health center has good access to essential drugs and medications. | 5 | 4 | 3 | 2 | 1 |
| 52 | My Rural Health Unit (RHU) has access to resources for health programs and projects. | 5 | 4 | 3 | 2 | 1 |
| 53 | I work with a competent LGU. | 5 | 4 | 3 | 2 | 1 |

Section IV: Local Environment Factors

*The following questions refer to your local or living conditions, this includes adequate housing, health care, schools for children, safety and security (i.e., psychosocial safety climate), basic utilities such as clean drinking water, electricity, and roads and transportation, acceptance by the community, and even social entertainment facilities while working as a DTTB. Please encircle the number that best fits your level of agreement with each statement, using a 5-point scale where 5=strongly agree, 4=agree, 3=neutral, 2=disagree, 1=strongly disagree.*

| No. | **To what extent do you agree with the following statements?** | 5 = Strongly Agree | 4 = Agree | 3 = Neutral | 2 = Disagree | 1 = Strongly disagree |
|---|---|---|---|---|---|---|
| 54 | I have access to clean running water at my workplace. | 5 | 4 | 3 | 2 | 1 |
| 55 | I have regular electricity at my workplace. | 5 | 4 | 3 | 2 | 1 |
| 56 | I have safe and efficient transportation to work. | 5 | 4 | 3 | 2 | 1 |
| 57 | I have access to clean running water at my accommodation. | 5 | 4 | 3 | 2 | 1 |
| 58 | I have regular electricity at my accommodation. | 5 | 4 | 3 | 2 | 1 |
| 59 | My accommodation has a comfortable place to sleep. | 5 | 4 | 3 | 2 | 1 |
| 60 | My accommodation has a clean toilet and shower. | 5 | 4 | 3 | 2 | 1 |
| 61 | Supplies for my personal needs are available in my area of assignment. | 5 | 4 | 3 | 2 | 1 |
| 62 | My area of assignment has sufficient options for leisure and entertainment. | 5 | 4 | 3 | 2 | 1 |

| | | | | | | |
|---|---|---|---|---|---|---|
| 63 | I feel safe in my area of assignment. | 5 | 4 | 3 | 2 | 1 |
| 64 | I consider myself a part of the community to which I am assigned. | 5 | 4 | 3 | 2 | 1 |

Section V: National Environment Factors

*The following questions refer to your compensation and financial incentives, as well as social unrest and conflict that impact rural health worker retention in the national environment. Please encircle the number that best fits your level of agreement with each statement, using a 5-point scale where 5=strongly agree, 4=agree, 3=neutral, 2=disagree, 1=strongly disagree.*

| No. | **To what extent do you agree with the following statements?** | 5 = Strongl y Agree | 4 = Agree | 3 = Neutral | 2 = Disagree | 1 = Strongly disagree |
|---|---|---|---|---|---|---|
| 65 | My salary is fair. | 5 | 4 | 3 | 2 | 1 |
| 66 | My benefit package (e.g. PhilHealth, GSIS, etc.) is fair. | 5 | 4 | 3 | 2 | 1 |
| 67 | My representation and travel allowances are fair. | 5 | 4 | 3 | 2 | 1 |
| 68 | My additional benefits from the LGU and DOH are fair. | 5 | 4 | 3 | 2 | 1 |
| 69 | My work is not affected by recent political conflict in the area | 5 | 4 | 3 | 2 | 1 |
| 70 | I was not threatened in any way during my stay in the area. | 5 | 4 | 3 | 2 | 1 |
| 71 | I consider myself safe from terrorism and other forms of violence. | 5 | 4 | 3 | 2 | 1 |

Section VI: International Environment Factors

*The following questions refer to your opportunities such as "higher rates of remuneration, more satisfying working conditions, a safer working environment and better educational and career development opportunities, as well as broader factors such as higher quality of life, freedom from political persecution, freedom of speech and educational opportunities for children" attract health workers to international destinations. Please encircle the number that best fits your level of agreement with each statement, using a 5-point scale where 5=strongly agree, 4=agree, 3=neutral, 2=disagree, 1=strongly disagree.*

| No. | **To what extent do you agree with the following statements?** | 5 = Strongly Agree | 4 = Agree | 3 = Neutral | 2 = Disagree | 1 = Strongly disagree |
|---|---|---|---|---|---|---|
| 72 | I am satisfied with my work as a DTTB here in the Philippines. | 5 | 4 | 3 | 2 | 1 |
| 73 | I am satisfied with my opportunities for career development here in the Philippines. | 5 | 4 | 3 | 2 | 1 |
| 74 | I am thinking about studying abroad after working as a DTTB | 5 | 4 | 3 | 2 | 1 |

| | | | | | | |
|---|---|---|---|---|---|---|
| 75 | I am thinking about working abroad after working as a DTTB | 5 | 4 | 3 | 2 | 1 |

Section VII: Degree of significance of factors that influence your decision to remain in your area of assignment after your term as DTTB.
*Please encircle the appropriate response.*

| No. | **If you were to decide to <u>remain in your municipality</u>, how important would the following factors be in that decision?** | 5 = Non-negotiable | 4 = Negotiable | 3 = May not be considered | 2 = Not important | 1 = No opinion |
|---|---|---|---|---|---|---|
| 76 | Good pay/allowances/benefits | 5 | 4 | 3 | 2 | 1 |
| 77 | Reasonable workload | 5 | 4 | 3 | 2 | 1 |
| 78 | Availability and access to supplies & equipment needed for regular RHU operations | 5 | 4 | 3 | 2 | 1 |
| 79 | Access to resources for health programs and projects | 5 | 4 | 3 | 2 | 1 |
| 80 | Local good governance | 5 | 4 | 3 | 2 | 1 |
| 81 | Assurance of continued support from the DOH | 5 | 4 | 3 | 2 | 1 |
| 82 | Available career opportunities | 5 | 4 | 3 | 2 | 1 |
| 83 | Good social relations in the workplace | 5 | 4 | 3 | 2 | 1 |
| 84 | Ease of management and supervision | 5 | 4 | 3 | 2 | 1 |
| 85 | Quality and availability of transportation | 5 | 4 | 3 | 2 | 1 |
| 86 | Running water and electricity at my accommodation | 5 | 4 | 3 | 2 | 1 |
| 87 | Running water and electricity at my workplace | 5 | 4 | 3 | 2 | 1 |
| 88 | Availability of communications services (internet, mobile phone signal, landline) | 5 | 4 | 3 | 2 | 1 |
| 89 | Quality and affordability of housing | 5 | 4 | 3 | 2 | 1 |
| 90 | Low cost of living | 5 | 4 | 3 | 2 | 1 |
| 91 | Safety of the community | 5 | 4 | 3 | 2 | 1 |
| 92 | Educational facilities for children | 5 | 4 | 3 | 2 | 1 |
| 93 | Access to higher education for myself | 5 | 4 | 3 | 2 | 1 |
| 94 | Distance of my workplace from my family | 5 | 4 | 3 | 2 | 1 |
| 95 | Distance of my workplace to a major city | 5 | 4 | 3 | 2 | 1 |

| | | | | | | |
|---|---|---|---|---|---|---|
| 96 | Opportunities for leisure, recreation, entertainment | 5 | 4 | 3 | 2 | 1 |
| 97 | Availability of business opportunities | 5 | 4 | 3 | 2 | 1 |
| 98 | Other: | 5 | 4 | 3 | 2 | 1 |
| 99 | Other: | 5 | 4 | 3 | 2 | 1 |

Section VIII: Future Plans

*Please encircle the appropriate response to the questions below.*

| No. | Questions | Please encircle your answers |
|---|---|---|
| 100 | Which of the following statements is true for you? | 1 = I plan to leave this job as soon as I can.<br>2 = I plan to seek another job once I finish my term as a DTTB.<br>3 = I plan to stay in the DTTB Program but in another area of assignment.<br>4 = I plan to remain in my current area of assignment after my term as a DTTB. |
| 101 | If you answered 4 in the previous statement and plan to remain in your current are of assignment after your term as a DTTB, how long do you plan to stay? | 1= I plan to stay in my area of assignment for □□ year/s.<br>2= I plan to stay in my area of assignment for good.<br>3= I do not know how long I will stay. |
| 102 | If you do not plan to stay in the DTTB Program or remain in your area of assignment which of the following statements best apply to you? | 1 = I plan to work as a clinician in the Philippines.<br>2 = I plan to work in the field of public health in the Philippines.<br>3 = I plan to switch to another job outside of the health sector, but within the Philippines.<br>4 = I plan to work outside the country. |

Section IX: Open-ended questions about your work as a DTTB.

103. Identify three things that you like about working as a DTTB:

a.
b.
c.

104. Identify three things you do not like about working as a DTTB:

a.
b.
c.

105. What would be the most important deciding factor for you to remain in your area of assignment after your term as a DTTB?

106. Do you have any suggestions on how to encourage DTTBs to choose to remain in their areas of assignment after graduating from the program?

107. Other comments:

**(End of the questionnaire)**
**Thank you for your cooperation!**
