# Supplementary material for: Factors affecting retention in the Philippine National Rural Physician Deployment Program from 2012 to 2019: a mixed methods study
Source: BMC Health Serv Res. 2021 Nov 5;21:1201. doi: 10.1186/s12913-021-07219-0 (PMC8571874; doi:10.1186/s12913-021-07219-0)
Supplement: Supplementary file 2 — Additional file 2. Interview Questions [file 12913_2021_7219_MOESM2_ESM.pdf]

Additional File 2 - Interview Questions

A. Individual Environmental Factors

1. What are your opinions on staying and working in a rural setting?
    a. How does working in a rural setting affect your relationships with your loved ones
    b. What are the most important challenges that you encountered while working in the rural areas and how do you cope up with it?
    c. What are the things that helped you adapt in your area?
2. What are your major stressors in your work as a DTTB?
    a. How do you cope up with these stressors?
    b. How do these things affect your work?
3. How does your family support you in your work as DTTB?
    a. What kind of support do you get from family or friends?
    b. How do you communicate with your family during your stay in your area?
    c. Are there any instances that you have felt homesick which may have affected your work?
    d. Are there experiences during your stay that have challenged your personal values, beliefs and spiritual motivations?

B. Work-related environment factors

1. Are you satisfied with your current job?
    a. How do you think has your job contributed to your career development as a doctor? Are there enough opportunities for career development?
    b. Do you think your current skills and experience match with your current job?
        - In what ways has your previous training help you to perform the work expected of you?
2. How does the government sectors (DOH staff, LGU officials, DOH regional office, DOH central office, municipal government) support you?
    a. What positive/negative working conditions have you encountered in your area? (poor hospital infrastructure, inadequate equipment, lack of medical supplies, staff shortages, lack of supervision, mentoring, supportive leadership, work environment, safety and security, workload)
    b. How has it affected your service to the community? Cite instances.

C. Local Environment factors

1. How do the living conditions in your area affect your decision to stay in the program?
    - Clean running water, electricity, transportation, cleanliness of the accommodation, options for leisure and entertainment
2. How has the community responded to your service?
    - Do you think your service is positively reciprocated by the community?
3. How has politics or other social conflicts affected your service in the community? Cite positive/negative ways.
    - Were there instances that your safety and security were threatened?

D. National Environment Factors

1. Do you think your current compensation and financial incentives equate for the amount of work you do?

a. Are there other modes of compensation you would like to request aside from benefit packages (PhilHealth, GSIS), representation and travel allowances?
b. Does this affect your morale to work or decision to stay in the program?

E. International Environment Factors

1. What opportunities abroad would make you want to work there?
    - What are your thoughts on career development opportunities abroad? Quality of life? Benefits?
